# 14-3-3ζ delivered by hepatocellular carcinoma-derived exosomes impaired anti-tumor function of tumor-infiltrating T lymphocytes

**DOI:** 10.1038/s41419-017-0180-7

**Published:** 2018-02-07

**Authors:** Xiaochen Wang, Haiyuan Shen, Guangyan Zhangyuan, Ruyi Huang, Wenjie Zhang, Qifeng He, Kangpeng Jin, Han Zhuo, Zechuan Zhang, Jincheng Wang, Beicheng Sun, Xiaojie Lu

**Affiliations:** 1 Liver Transplantation Center of the First Affiliated Hospital, Nanjing Medical University, Nanjing, Jiangsu Province 210029 China; 2 Department of Hepatobiliary Surgery, The Affiliated Drum Tower Hospital of Nanjing University Medical School, Nanjing, Jiangsu Province 210009 China

## Abstract

Increasing evidence shows that the anti-tumor functions of tumor-infiltrating T lymphocytes (TILs) were inhibited significantly, but the underlying mechanisms remain not fully understood. In this study, we found that 14-3-3ζ expression was up-regulated in hepatocellular carcinoma (HCC) cells and in TILs. TILs with 14-3-3ζ high-expression (14-3-3ζ^high^) exhibited impaired activation (CD69), proliferation (Ki67) and anti-tumor functions compared to 14-3-3ζ low expression (14-3-3ζ^low^) TILs. Flow cytometry assay showed that compared with 14-3-3ζ^low^ CD8^+^T cells, 14-3-3ζ^high^ ones exhibited higher frequency of exhausted phenotypes as measured by inhibitory receptors such as PD-1, TIM-3, LAG3, and CTLA-4. 14-3-3ζ overexpression inhibited the activity and proliferation of peripheral blood CD3^+^ T cells, deviated the differentiation of naive T cells from effector T cells to regulatory T cells. Moreover, we found that 14-3-3ζ expression levels in TILs correlated positively with those in HCC cells. Naive T cells co-cultured with HCC cells or the visible components of culture medium of HCC cells exhibited increased 14-3-3ζ expression. Stochastic optical reconstruction microscopy (STORM) and confocal assay showed that 14-3-3ζ-containing exosomes derived from HCC cells could be swallowed by T cells, suggesting that 14-3-3ζ might be transmitted from HCC cells to TILs at least partially through exosomes. In conclusion, our study for the first time demonstrated that 14-3-3ζ is up-regulated in and inhibited the anti-tumor functions of tumor-infiltrating T cells in HCC microenvironment and that 14-3-3ζ might be transmitted from HCC cells to T cells at least partially through exosomes.

## Facts

- 14-3-3ζ was highly expressed in HCC cells and in TILs.
- 14-3-3ζ impaired the anti-tumor activity of TILs in HCC.
- 14-3-3ζ was associated with T cells exhaustion.
- 14-3-3ζ could be transmitted from HCC cells to TILs at least partially through exosomes.

Hepatocellular carcinoma (HCC) accounts for ninety percent of primary liver cancer, which is the fifth most common cancer and the second leading cancer death worldwide^1^. Increasing evidence has shown that in cancer immune microenvironment (IME), there is an increase in the number of immunosuppressive cells and in the meanwhile a decrease in the number and function of anti-cancer immunocytes^2,3^. Lymphocytes in peripheral blood mononuclear cells (PBMC) that infiltrate into tumor microenvironment are called tumor infiltrating lymphocytes, which are the main component of tumor IME^4,5^. Previous studies in HCC have demonstrated that the anti-tumor effects of T cells in tumor microenvironment decreased significantly, but the underlying mechanisms remain to be fully elucidated^6,7^.

14-3-3ζ, also called 14-3-3 protein zeta, has been reported to be highly expressed in HCC, and to promote the proliferation and epithelial-mesenchymal transition (EMT) of HCC cells^8,9^. Moreover, clinical study has shown that high expression of 14-3-3ζ in patients with HCC correlated with poor survival^8,10^. However the roles of 14-3-3ζ in tumor infiltrating T lymphocytes (TILs) have rarely been studied. Our preliminary data for the first time showed that the expression of 14-3-3ζ in HCC cells correlated significantly with that in TILs in HCC, however, the underlying mechanisms are obscure.

Exosome is a kind of tiny membrane vesicles that contains small molecules such as RNA, DNA and protein. It is exported by a diversity of cells and released into the microenvironment, which can then be up-taken by other cells^11,12^. Former studies have reported exosome as a transporter of diverse molecules associated with drug-resistance and metastasis in cancer^13–15^. So, we speculate that 14-3-3ζ might be delivered by exosomes from HCC cells to  TILs and may affect the functionality of the latter. To testify our speculation, we designed this study to explore whether 14-3-3ζ is delivered from HCC cells to TILs through exosomes and to investigate the functional roles of 14-3-3ζ in TILs, in the hope of providing a new avenue to understand the mechanisms of dysfunctionality of T cells in HCC microenvironment.

## Results

### 14-3-3ζ was highly expressed in HCC cells and in TILs

**Fig. 1:**
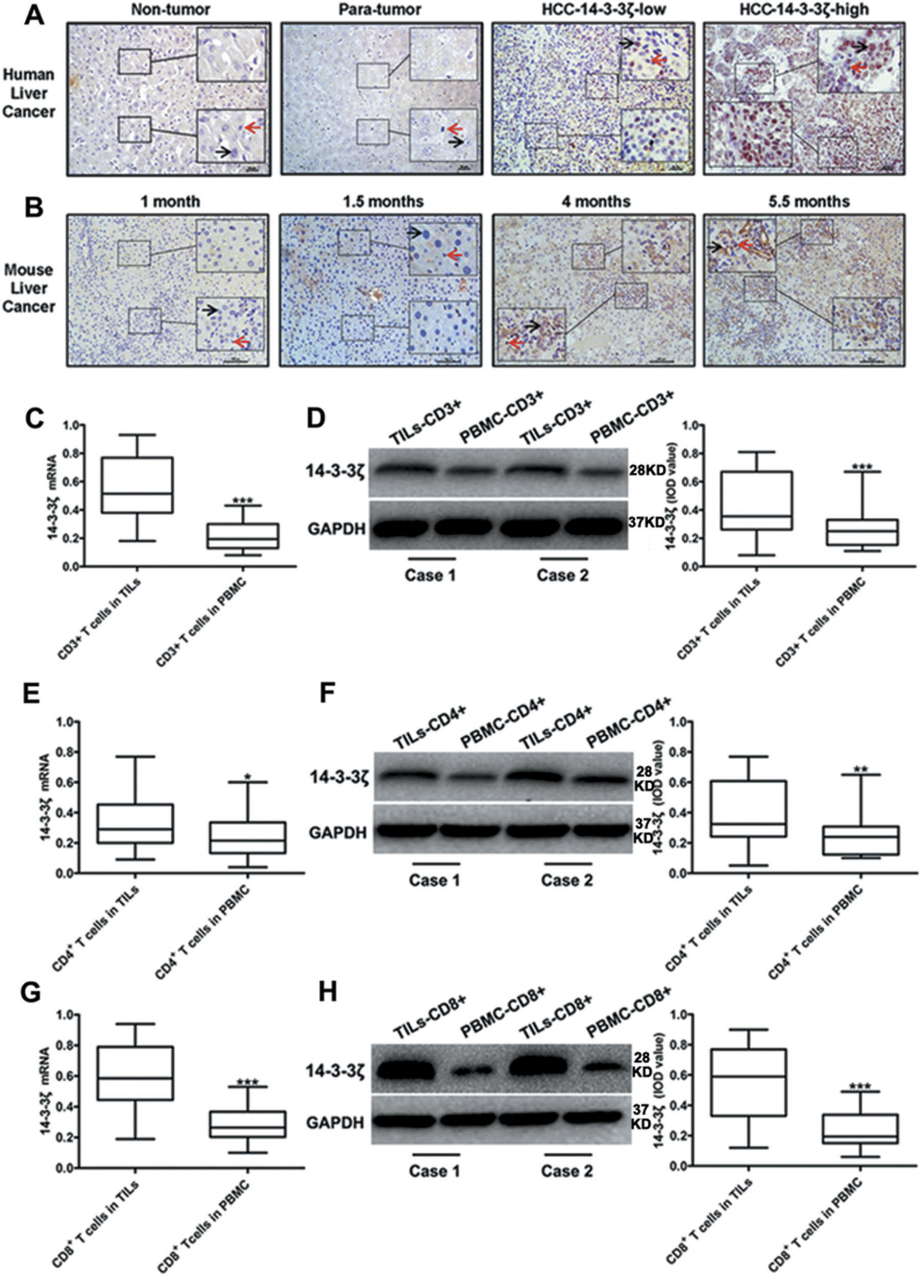
The expression of 14-3-3ζ in HCC cells and TILs in HCC. **a** Representative results of IHC staining of 14-3-3ζ in normal liver tissues (*n* = 10), paracancerous tissues (*n* = 28) and HCC tissues (*n* = 28). 14-3-3ζ was stained in brown and nuclear was stained in blue. (Black arrow: hepatocyte/HCC cells; Red arrow: TILs; Scale = 50 µm, ×200 and ×400). **b** Representative IHC staining in liver tissues from chronic inflammation-related mice HCC model. 14-3-3ζ and nuclear were stained in brown and blue respectively. 1 month, 2.5 months, 4 months and 5.5 months refer to ages of mice. (Black arrow: hepatocyte/HCC cells; Red arrow: TILs; Scale = 100 µm, ×200 and ×400). **c**, **d** mRNA (**c**) and protein (**d**) levels of 14-3-3ζ in CD3^+^ T cells from TILs and PBMC respectively. **e**, **f** mRNA (**e**) and protein (**f**) levels of 14-3-3ζ in CD4^+^ T cells from TILs and PBMC respectively. **g**, **h** mRNA (**g**) and protein (**h**) levels of 14-3-3ζ in CD8^+^ T cells from TILs and PBMC respectively. Data were presented as box plots. Box plot explanation: upper horizontal line of box, 75th percentile; lower horizontal line of box, 25th percentile; horizontal bar within box, median; upper horizontal bar outside box, 95th percentile; lower horizontal bar outside box, 5th percentile. All experiments were performed in independent triplicate. (*P < 0.05, **P < 0.01, ***P < 0.001)

Analysis based on Oncomine database of expression profiles with clinical cancer samples suggested that 14-3-3ζ mRNA level was significantly higher in HCC tissues than in cancer surrounding tissues (Fig. S1). In line with this, our results showed that 14-3-3ζ was highly expressed in HCC tissues compared to paracancerous tissues and normal liver tissues (Fig. 1a). Then, we constructed an inflammation-related HCC mouse model, positive staining of 14-3-3ζ cannot be observed until tumor was developed (Fig. 1b; Fig. S2).

Moreover, mRNA and protein expression levels of 14-3-3ζ in TILs were significantly increased relative to those in peripheral blood T cells (PBTC) (Fig. 1c, d). Next, we assayed the expression levels of 14-3-3ζ in CD4^+^ and CD8^+^ T cells separated from TILs or PBMC of HCC patients, respectively, the results of which showed that 14-3-3ζ protein and mRNA levels increased significantly in CD4^+^ and CD8^+^ T cells isolated from TILs compared to those from PBMC (Fig. 1e–h). In sum, 14-3-3ζ was highly expressed only in cancer cells and T cells in HCC microenvironment.

### 14-3-3ζ impaired the functions, proliferation and activation of T cells and regulate their differentiation

**Fig. 2:**
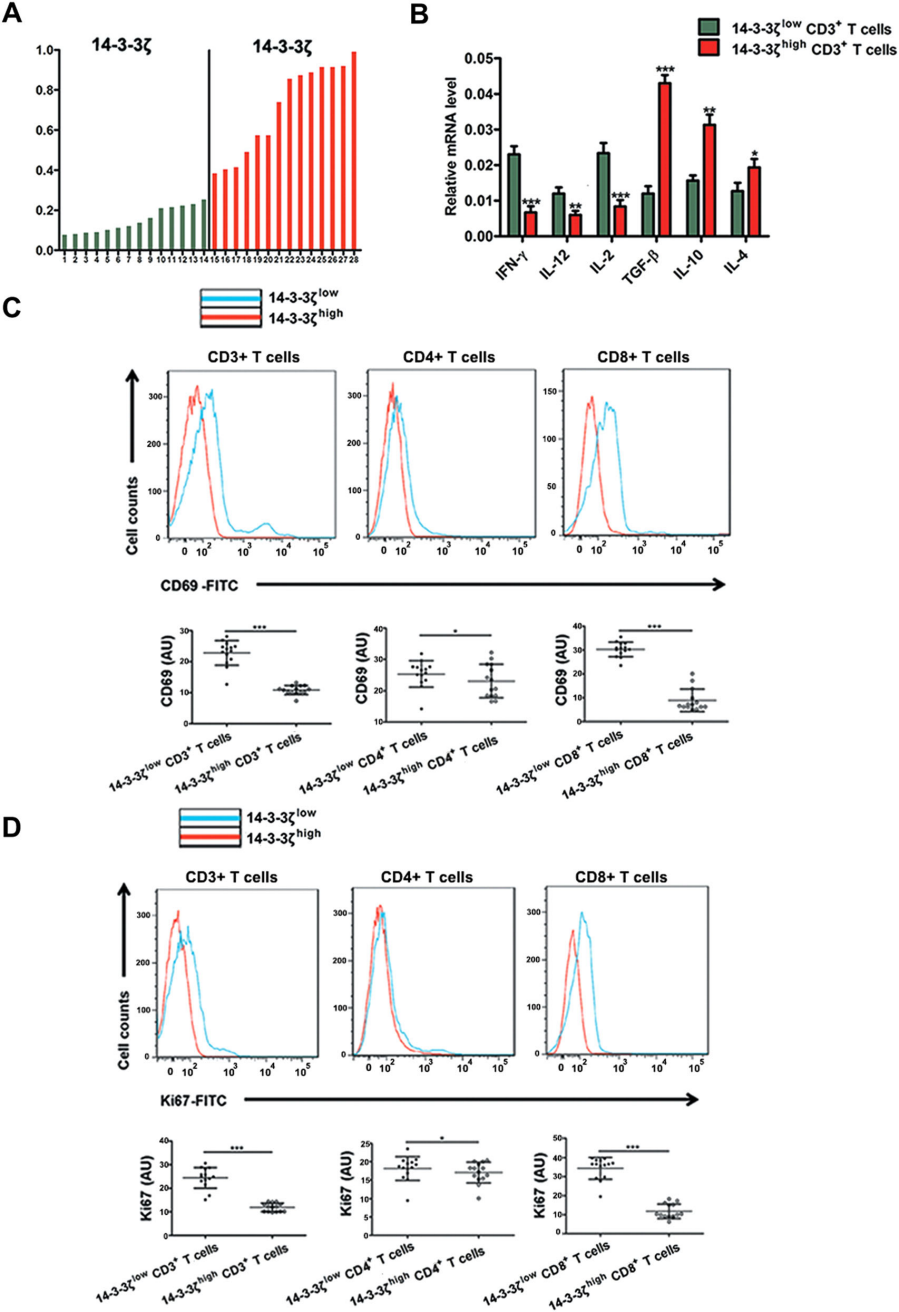
Cytokine Secretion, cell viability and proliferation of T cells from 14-3-3ζ^high^ and 14-3-3ζ^low^ group. **a** Totally 28 HCC tissues were divided into 14-3-3ζ^low^ group and 14-3-3ζ^high^ group according to the expression level of 14-3-3ζ in CD3^+^ T cells assay by western blot. A total of 28 tumor tissues with adequate size which can could satisfy the demand for cells isolation were selected for the analysis of relative parts. The media 14-3-3ζ expression level (Relative IOD value of 14-3-3ζ assayed by western blot compared to that of GAPDH). **b** mRNA expression levels of IFN-γ, IL-12, IL-2, TGF-β, IL-10, and IL-4 were assayed in CD3^+^ T cells from 14-3-3ζ^high^ (*n* = 14) and 14-3-3ζ^low^ (*n* = 14) group, respectively. The experiments were performed in independent triplicate. **c** Flow cytometry assays of CD69 levels in CD3^+^, CD4^+^, and CD8^+^ T cells from 14-3-3ζ^high^ (*n* = 14) and 14-3-3ζ^low^ (*n* = 14) groups, respectively. **d** Flow cytometry assays of Ki67 levels in CD3^+^, CD4^+^, and CD8^+^ T cells from 14-3-3ζ^high^ (*n* = 14) and 14-3-3ζ^low^ (*n* = 14) groups respectively. Data were presented as the mean ± SEM (*P < 0.05, **P < 0.01, ***P < 0.001)

A total of 28 HCC tissues were subjected to immunohistochemistry (IHC) of 14-3-3ζ and were divided into 14-3-3ζ^high^ group (*n* = 14) and 14-3-3ζ^low^ group (*n* = 14) (cut-off by median 14-3-3ζ expression level—relative integrated optical density (IOD) value of 14-3-3ζ assayed by western blot compared to that of GAPDH) (Fig. 2a). Analysis on clinicopathological features indicated that high-expression of 14-3-3ζ was associated with larger tumor size, poor tumor differentiation and terminal TNM stage (Table 1). The mRNA levels of the inflammatory cytokines (IFN-γ, IL-12, IL-2) and anti-inflammatory cytokines (TGF-β, IL-10, and IL-4) were assayed in  TILs isolated from both groups (14-3-3ζ^high^ and 14-3-3ζ^low^). Interestingly, compared with 14-3-3ζ^high^  TILs, 14-3-3ζ^low^  TILs expressed much higher levels of inflammatory cytokines but lower levels of anti-inflammatory cytokines (Fig. 2b).

**Table 1:** Patient cohort description

Feather	Number	High	Low	*P* value
All cases	28	14	14	
Age(years)				0.699
<60	11	5	6	
≥60	17	9	8	
Gender				0.541
Male	25	13	12	
Female	3	1	2	
Tumor size (cm)				**0.004**
<3	8	2	6	
3-5	6	2	4	
>5	14	10	4	
Differentiation grade				**<0.001**
Well	3	0	3	
Moderate	14	5	9	
Poorly	11	9	2	
Tumor number				0.445
Solitary	12	5	7	
Multiple	16	9	7	
Tumor capsular				0.309
Incomplete	1	1	0	
Complete	27	13	14	
TNM stage				**0.001**
I	4	1	3	
II	13	5	8	
III	11	8	3	

P-value in bold indicates statistically significant

**Fig. 3:**
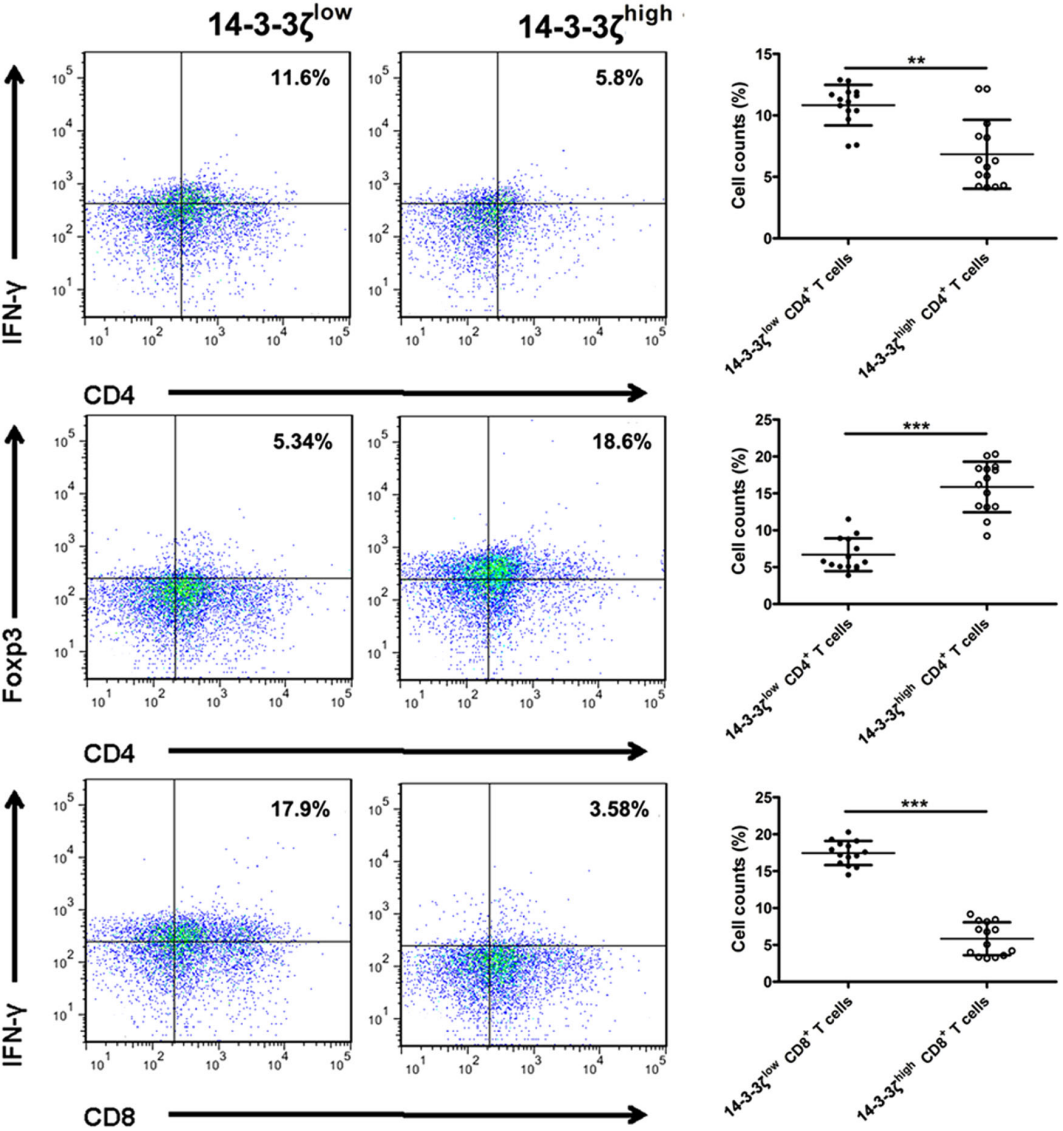
Anti-tumor functions of T cells of 14-3-3ζ^high^ and 14-3-3ζ^low^ goups in HCC. Flow cytometry analyses of the proportions of CD4^+^IFN-γ^+^, CD4^+^Foxp3^+^ and CD8^+^IFN-γ^+^ T cells among tumor-infiltrating CD3^+^ T cells from 14-3-3ζ^high^ (*n* = 14) and 14-3-3ζ^low^ (*n* = 14) groups, respectively. Data were presented as the mean ± SEM (**P < 0.01, ***P < 0.001)

CD3^+^ T cells, CD4^+^ T cells and CD8^+^ T cells isolated from HCC tissues (Fig. S3) in both groups (14-3-3ζ^high^ and 14-3-3ζ^low^) were stimulated with anti-CD3/CD28 beads for three consecutive days and then subjected to flow cytometry (FCM) to measure the percentage of Ki67^+^ cells and CD69^+^ cells. The results showed that CD3^+^ T cells, CD4^+^ T cells and CD8^+^ T cells isolated from 14-3-3ζ^high^ HCC tissue displayed decreased activity and proliferation ability compared with those from 14-3-3ζ^low^ HCC tissue (Fig. 2c, d). Subsequently, the percentages of effector T cells (T_eff_) and regulatory T cells (T_reg_) in CD3^+^ T cells were assayed by FCM, the results of which showed that the percentage of T_eff_ decreased whereas that of T_reg_ increased in 14-3-3ζ^high^ group versus 14-3-3ζ^low^ group (Fig. 3).

**Fig. 4:**
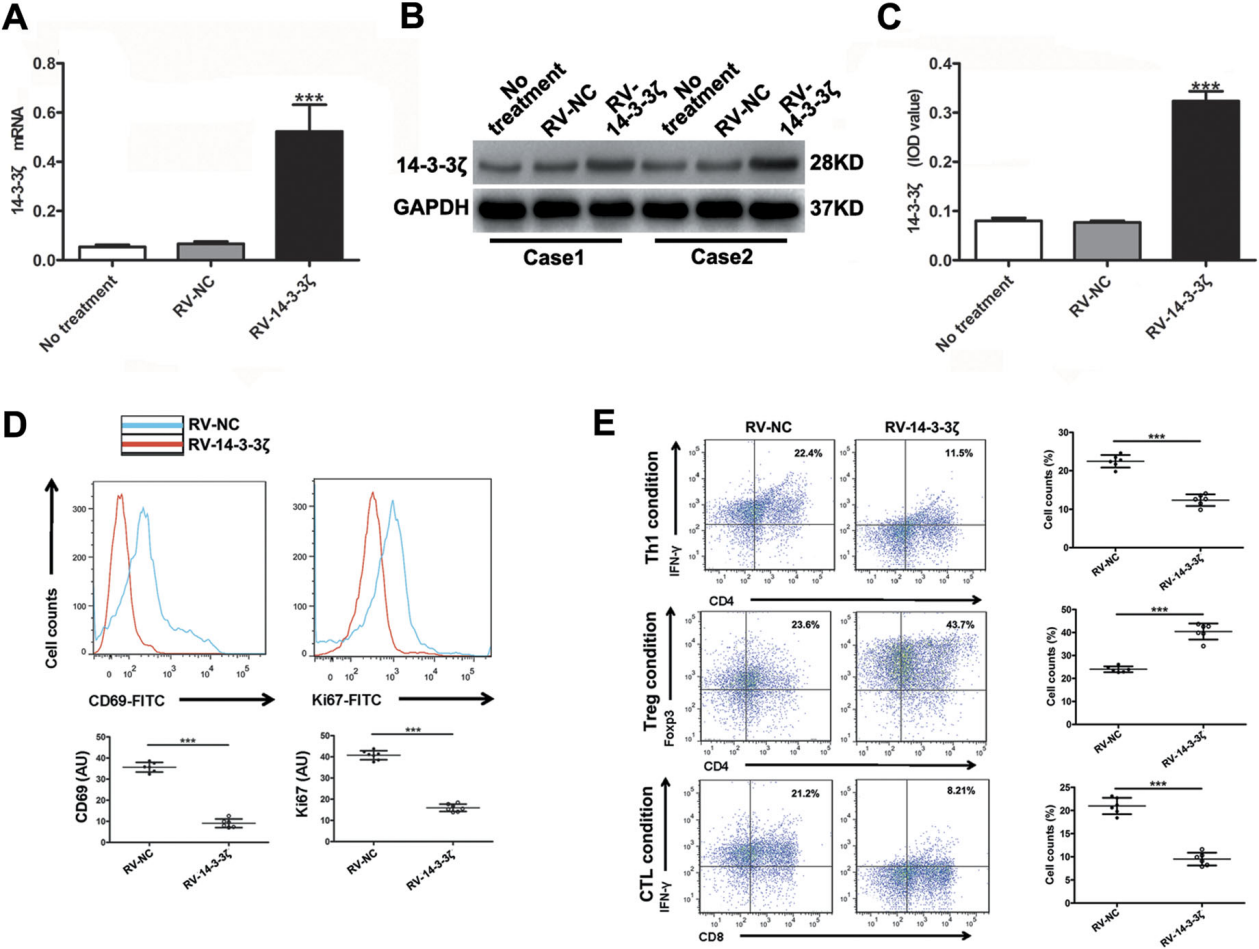
14–3–3ζ overexpression inhibited the viability and proliferation of T cells and deviated the differentiation of naive T cell from effector T cells (T_eff_) to regulatory T cells (T_reg_). **a** Naive T cells were transfected with empty retro-virus (RV-NC) or overexpressing 14-3-3ζ retro-virus (RV-14-3-3ζ) then the mRNA level of 14-3-3ζ was detected by real-time PCR (*n* = 6). **b**, **c** Naive T cells were transfected with RV-NC or RV-14-3-3ζ then the protein level of 14-3-3ζ was detected by western blot (*n* = 6). Data were presented as the mean ± SEM. All experiments were performed in independent triplicate. **d** Flow cytometry assay of CD69 and Ki67 in CD3^+^ T cells transfected with empty retrovirus (RV-NC) (*n* = 6) or 14-3-3ζ-expressing RV (RV-14-3-3ζ) (*n* = 6). **e** Flow cytometric analyses of the proportions of polarizing T cells (CD4^+^IFN-γ^+^, CD4^+^Foxp3^+^, and CD8^+^IFN-γ^+^ T cells) induced from naive T cells transfected with RV-NC (*n* = 6) or RV-14-3-3ζ (*n* = 6) Data were presented as the mean ± SEM (*** *P* < 0.001)

To further investigate the effects of 14-3-3ζ on the activation, proliferation and differentiation of T cells, we transfected naive T cells (CD45RA^+^CD197^+^) isolated from human PBMC with RV-NC (retro-virus packaged with empty plasmid) or RV-14-3-3ζ (retro-virus packaged with 14-3-3ζ overexpressing plasmid) (Fig. 4a–c). Subsequently, part of these cells were stimulated with anti-CD3/CD28 beads for three days and then subjected to FCM to measure the percentages of CD69^+^ cells and Ki67^+^ cells (Fig. 4d); and the other part of these cells were stimulated with IL-12 (10 ng/ml), IL-2 (10 ng/ml) or TGF-β (10 ng/ml) respectively for 72 h and then subjected to FCM to assay CD4, CD8, Foxp3, and IFN-γ. We found that overexpression of 14-3-3ζ significantly inhibited the activation and proliferation of T cells and deviated the differentiation of naive T cells from T_eff_ to T_reg_ cells (Fig. 4e), suggesting that 14-3-3ζ regulates the activation, proliferation, and differentiation of T cells.

### 14-3-3ζ high expression correlate with T cells exhaustion

**Fig. 5:**
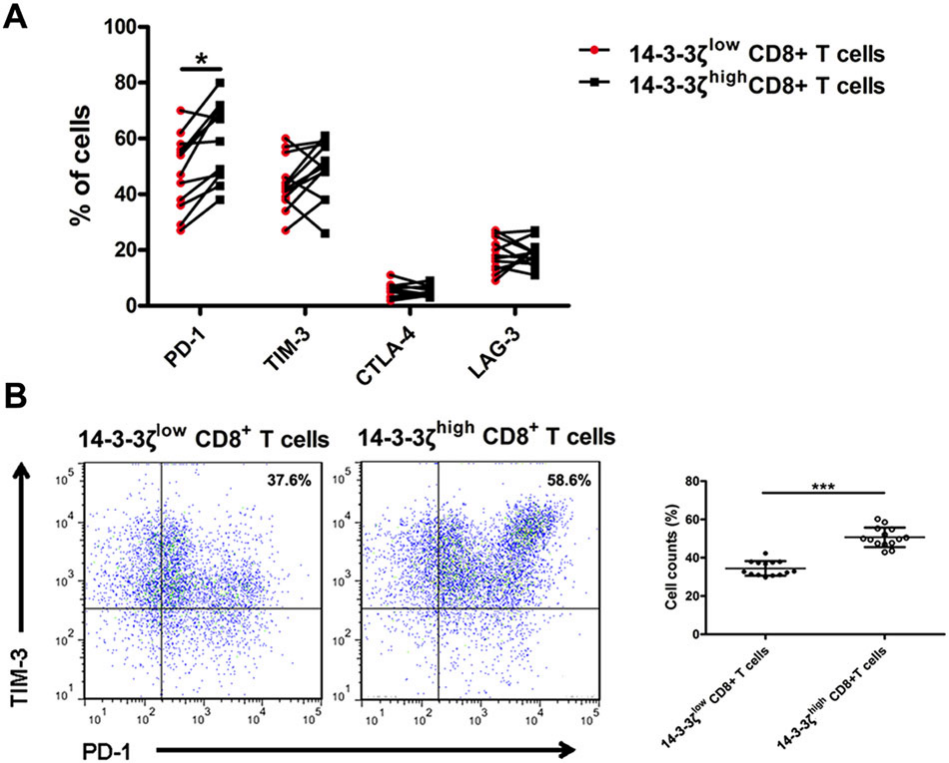
Detection of the expression levels of inhibitory receptors on CD8^+^ T cells from 14-3-3ζ^high^ and 14-3-3ζ^low^ group. **a** Flow cytometry assay of PD-1, TIM-3, CTLA-4 and LAG3 in 14-3-3ζ^high^ CD8^+^ T cells (*n* = 14) and 14-3-3ζ^low^ CD8^+^ T cells (*n* = 14). **b** Flow cytometry assay of PD-1^+^ TIM-3^+^ cells in 14-3-3ζ^high^ CD8^+^ T cells (*n* = 14) and 14-3-3ζ^low^ CD8^+^ T cells (*n* = 14). Data were presented as the mean ± SEM (*P < 0.05, ***P < 0.001)

Mounting evidence has demonstrated that T cell exhaustion is one of the major mechanisms of cancer immune evasion. As PD-1, TIM-3, LAG3 and CTLA-4 are well-established T cell exhaustion markers, we assayed these markers in CD8^+^ T cells isolated from TILs in 14-3-3ζ^high^ group and in 14-3-3ζ^low^ group respectively. The results showed that there was a significantly higher percentages of PD-1^+^ T cells and PD-1^+^TIM-3^+^ T cells in 14-3-3ζ^high^ CD8^+^ T versus 14-3-3ζ^low^ CD8^+^ T cells (Fig. 5a, b), indicating that 14-3-3ζ high expression correlated significantly with an exhausted phenotype of T cells.

### 14-3-3ζ was transmitted from HCC cells to TILs delivered by exosomes

**Fig. 6:**
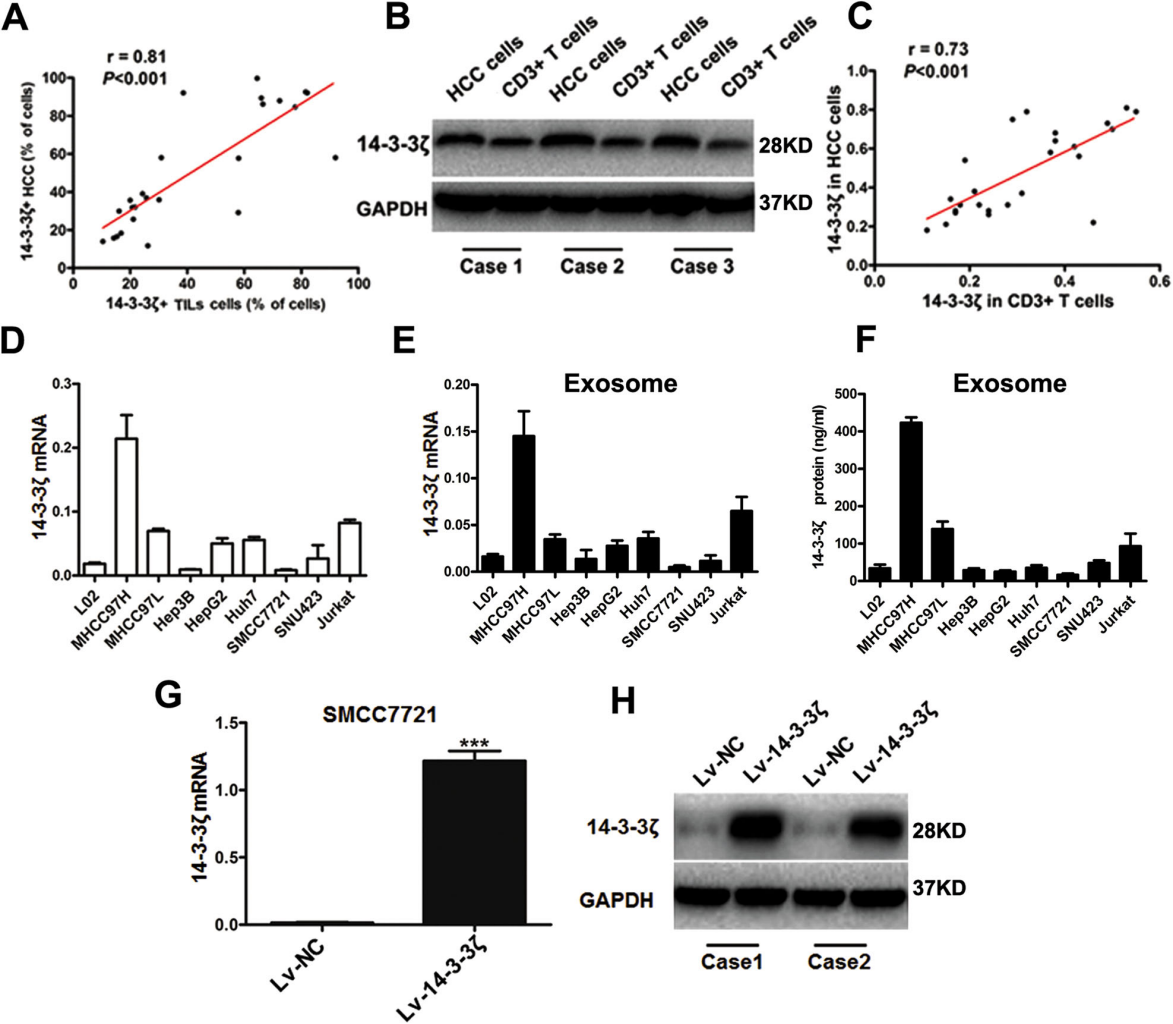
14–3–3ζ was detected in T cells and HCC cells. **a** Correlation between the percentages of 14-3-3ζ-positive HCC cells and those of 14-3-3ζ-positive TILs. **b**, **c** Left panel: Semiquantitative assay by western blot of the 14-3-3ζ expression in HCC cells and tumor-infiltrating CD3 + T cells (*n* = 25). Right panel: Correlation between the expression levels of 14-3-3ζ in HCC cells and tumor-infiltrating CD3^+^ T cells (*n* = 25). **d** The mRNA level of 14-3-3ζ was assayed in diverse HCC cell lines. (**e**) The mRNA level of 14-3-3ζ was assayed in exosome isolated from diverse HCC cell lines. **f** The protein level of 14-3-3ζ was assayed by ELISA assay in exosome isolated from diverse HCC cell lines. **g** SMCC7721 cells were transfected with empty lenti-virus (Lv-NC) or overexpressing 14-3-3ζ lenti-virus (Lv-14-3-3ζ) then the mRNA level of 14-3-3ζ was detected by real-time PCR. **h** SMCC7721 cells were transfected with Lv-NC or Lv-14-3-3ζ then the protein level of 14-3-3ζ was detected by western blot. Data were presented as the mean ± SEM. All experiments were performed in independent triplicate. (***P < 0.001)

IHC staining of HCC tissues and TILs showed that the percentage of 14-3-3ζ positive TILs (versus all TILs) positively correlated with that in HCC cells (versus all liver cells) (Fig. 6a). Real-time PCR and western blot assay in HCC cells and TILs separated from HCC tissues indicated that the protein level rather than the mRNA level of 14-3-3ζ in HCC cells correlated well with those in  TILs, (Fig. 6b, c, Fig. S4).

**Fig. 7:**
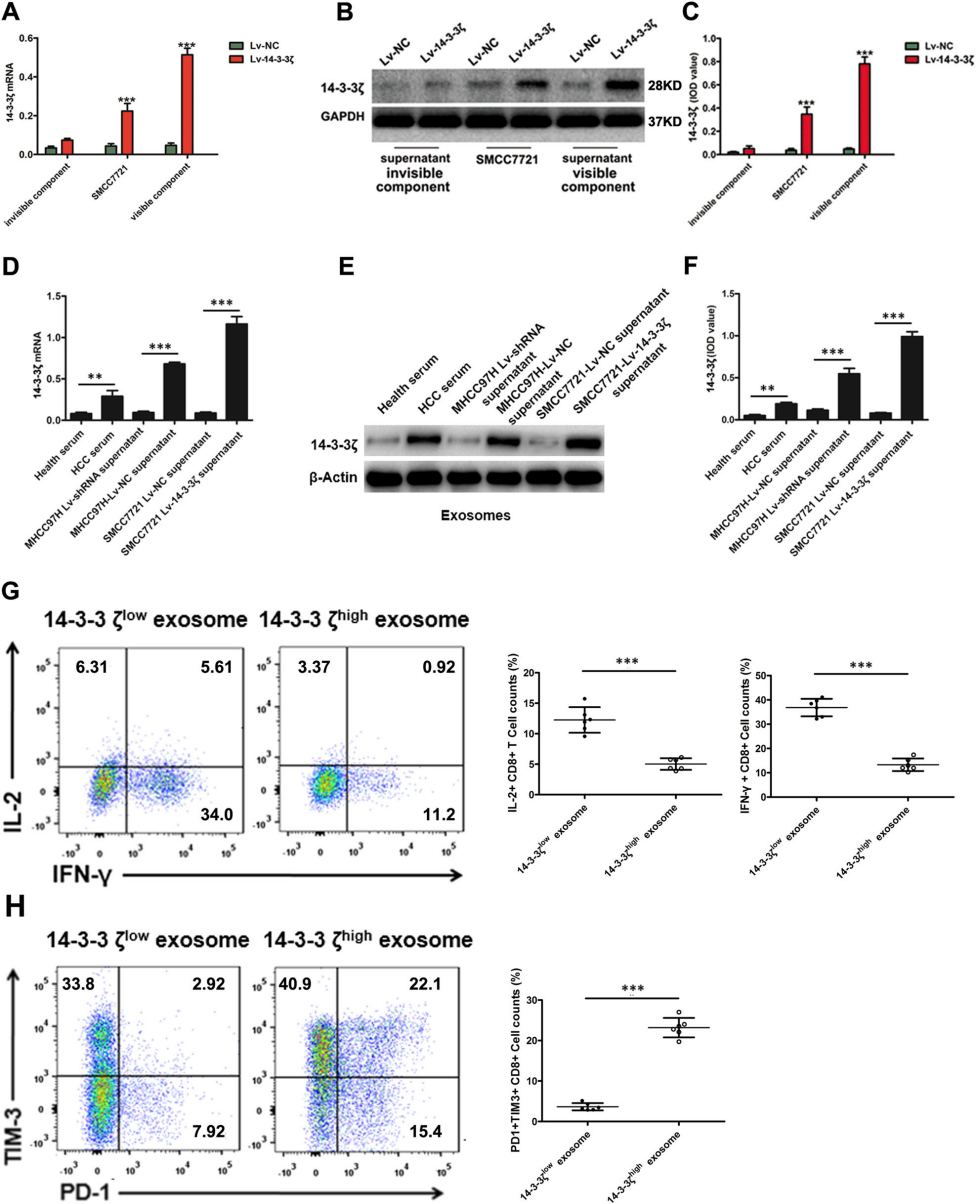
14–3–3ζ was detected in co-cultured naive T cells and isolated exosomes. **a** The mRNA level of T cells was detected by real-time PCR in naive T cells co-cultured with SMCC7721 transfected with Lv-NC or Lv-14-3-3ζ, the invisible or visible components from the culture supernatant of SMCC7721-Lv-NC and SMCC7721-Lv-14-3-3ζ. Data were presented as the mean ± SEM. All experiments were performed in independent triplicate. **b**, **c** Expression levels of 14-3-3ζ detected by western blot in naive T cells co-cultured with SMCC7721 transfected with empty lenti-virus (Lv-NC) or 14-3-3ζ-expressing lenti-virus (Lv-14-3-3ζ), as well as with the invisible or visible components from the culture supernatant of SMCC7721-Lv-NC and SMCC7721-Lv-14-3-3ζ (*n* = 6), respectively. Data were presented as the mean ± SEM. **d** The mRNA level of 14-3-3ζ was detected by real-time PCR in exosomes collected from serum of healthy control and HCC patients, the supernatant of MHCC97H-Lv-NC, MHCC97H-Lv-shRNA, SMCC7721-Lv-NC and SMCC7721-Lv-14-3-3ζ. **e**, **f** The protein level of 14-3-3ζ was detected by western blot in exosomes collected from serum of healthy control and HCC patients, the supernatant of MHCC97H-Lv-NC, MHCC97H-Lv-shRNA, SMCC7721-Lv-NC and SMCC7721-Lv-14-3-3ζ. **g**, **h** Flowcytometry analysis showed naive T cells co-cultured with 14-3-3ζ^high^ exosomes produced lower level of IL-2 and IFN-γ, and showed higher level of PD-1 and TIM-3 expression than those co-cultured with 14-3-3ζ^low^ exosomes. Data were presented as the mean ± SEM. All experiments were performed in independent triplicate. (***P < 0.001)

To investigate the mechanisms underlying the correlation between the expression levels of 14-3-3ζ in TILs and in HCC cells, we transfected SMCC7721 cells (Fig. 6d), which have the low level of 14-3-3ζ expression in their exosome (Fig. 6e, f), with Lv-NC (lenti-virus packaged with empty plasmid) or Lv-14-3-3ζ (lenti-virus packaged with 14-3-3ζ overexpressing plasmid). Then we co-cultured naive T cells (after stimulation with CD3/CD28 beads for 3 days) with Lv-14-3-3ζ-transfected SMCC7721 cells, Lv-NC-transfected SMCC7721 cells (Fig. 6g, h, Fig. S5), visible components or invisible components of culture medium of these cells. The results showed that 14-3-3ζ expression levels were increased in naive T cells co-cultured with Lv-14-3-3ζ-transfected SMCC7721 cells and with visible components of cell culture medium of Lv-14-3-3ζ-transfected SMCC7721 cells (Fig. 7a–c). These results indicated that 14-3-3ζ-overexpressing HCC cells or their visible components of cell culture medium is responsible for the transmission of 14-3-3ζ from HCC cells to T cells.

**Fig. 8:**
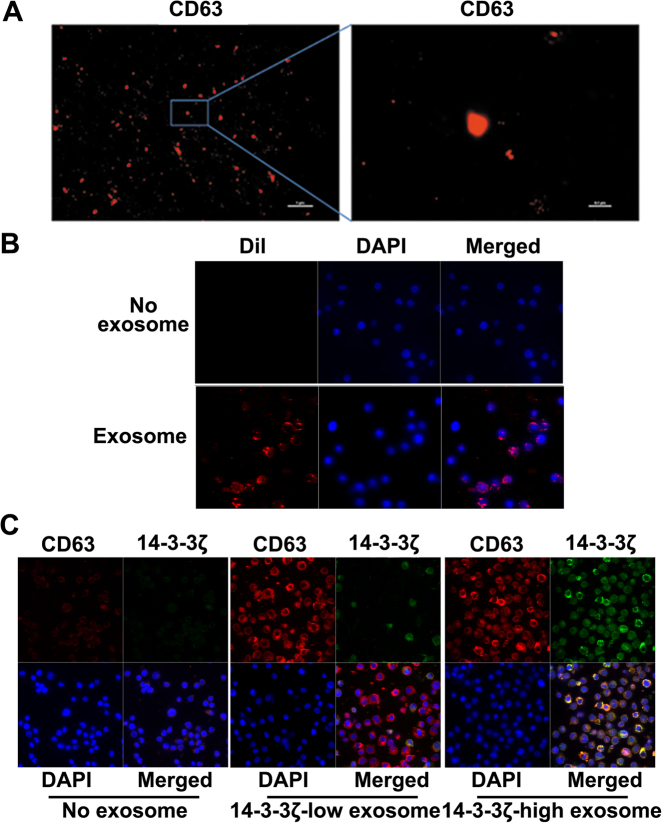
Increased 14-3-3ζ expression in T cells mediated by HCC cell-derived exosomes. **a** Exosomes stained with anti-human CD63 were detected by STORM (Scale: left panel, 1 µm; right panel: 0.1 µm) (*n* = 6). **b** Exosomes stained with dye for membrane (Dil, red) and subsequently co-cultured with naive T cells (blue indicates nuclear staining with DAPI, detected at one hour during co-culture by laser con-focal assay). Upper panel: Negative control; Lower panel: Added with exosome collected from HCC serum (*n* = 6). **c** Exosomes (14-3-3ζ^low^ or 14-3-3ζ^high^) were stained with anti-human CD63 (red) and anti-14-3-3ζ(green) and subsequently co-cultured with naive T cells (blue indicates nuclear stained with DAPI, detected at one hour post co-culture by laser con-focal assay). Addition of exosomes collected from HCC serum (*n* = 6)

As the visible components of cell culture medium were mainly composed of exosomes, we detected the level of 14-3-3ζ in exosomes isolated respectively from the serum of healthy individuals, serum of HCC patients, cell culture medium of MHCC97H cells (expressing relative higher level of 14-3-3ζ), Lv-shRNA transfected MHCC97H cells (Fig. 6d), SMCC7721 cells and Lv-14-3-3ζ transfected SMCC7721 cells. We found that the protein and mRNA levels of 14-3-3ζ were significantly higher in exosomes isolated from HCC serum compared to those from healthy serum. We found that in exosomes derived from 14-3-3ζ-overexpressing cells, the level of 14-3-3ζ is higher (Fig. 7d–f), which was in accordance with the former study^16^. We also found that naive T cells co-cultured with 14-3-3ζ^high^ exosomes produced lower level of IL-2 and IFN-γ, and showed higher level of PD-1 and TIM-3 expression than those co-cultured with 14-3-3ζ^low^ exosomes (Fig. 7g, h). Immunofluorescence staining assay indicated 14-3-3ζ can transfer into T cells, and moreover, we observed that 14-3-3ζ-containing exosomes exported by HCC cells can be swallowed by T cells. (Fig. 8a–d).

These results suggested that 14-3-3ζ could be transmitted from HCC cells to  TILs via exosomes.

## Discussion

In this study, we found that in HCC microenvironment,  TILs with 14-3-3ζ high expression presented impaired anti-tumor function compared to those with 14-3-3ζ low expression. Overexpression of 14-3-3ζ deviated the differentiation of T cells from effective ones to regulatory ones. What’s more, we found that up-regulation of 14-3-3ζ in  TILs was at least partially attributable to the up-take of 14-3-3ζ-containing exosomes exported from HCC cells.

Previous studies have suggested the oncogenic roles of 14-3-3ζ in diverse cancer types including HCC^17,18^. For example, 14-3-3ζ was upregulated in HCC and can promote HCC invasion and metastasis through binding to AXL (activity of the receptor tyrosine kinase Axl)^9^ or through interacting with αB-crystallin mediated by TGF-β and ERK signaling pathways^8^. However, to the best of our knowledge, the roles of 14-3-3ζ in T cell function were still not reported. Chimen, et, al. reported that PEPITEM, a peptide derived from 14-3-3ζ, inhibited T cell trafficking and thus reduce T cell recruitment into inflammation sites during chronic inflammation^19^, which suggest that 14-3-3ζ may affect T cell immunity in some way.

Our study for the first time found that the protein expression levels of 14-3-3ζ in HCC cells correlated positively with that in  TILs, and that the expression levels of 14-3-3ζ in  TILs of HCC were significantly higher than those in PBTC. To explore the effects of 14-3-3ζ expression levels on  TILs function, we assayed the activity and functionality of  TILs in 14-3-3ζ^high^ group and 14-3-3ζ^low^ group. The results showed that the activation and anti-tumor activity of  TILs in 14-3-3ζ^high^ group were significantly impaired compared to those in 14-3-3ζ^low^ group, as evidenced by decrease excretion of cytotoxic cytokines. As CD8^+^ T cells are the major anti-tumor effector cells in  TILs^20–22^, we assayed the exhaustion markers (PD-1, TIM-3, LAG3, CTLA-4)^23–25^ in CD8^+^ T cells isolated from 14-3-3ζ^high^ and from 14-3-3ζ^low^ group. The results showed increased expression of T cell exhaustion markers (PD-1^+^TIM-3^+^)^26,27^ in CD8^+^ T cells from 14-3-3ζ^high^ group as compared to those from 14-3-3ζ^low^ group, suggesting that 14-3-3ζ may contribute to CD8^+^ T cell exhaustion. Overexpression of 14-3-3ζ decreased the activation and proliferation of naive T cells and deviate the differentiation of the latter from effector T cells to regulatory T cells (Treg). These findings as a whole point to the conclusion that 14-3-3ζ impaired the anti-tumor activity of  TILs in HCC.

As 14-3-3ζ expression in HCC cells correlated positively with that in  TILs and the levels of 14-3-3ζ in  TILs were significantly higher than those in PBTC, we speculated that the expression of 14-3-3ζ in  TILs might be influenced by 14-3-3ζ in HCC cells through mechanisms such as direct contact transmission or indirect communications mediated by certain vehicles in the interstitial fluid like exosomes^28,29^.

To test our speculation, we co-cultured naive T cells respectively with HCC cells, visible components and invisible components of cell culture medium of 14-3-3ζ-overexpressing HCC cells. It has been previously reported that stromal cells uptake 14-3-3-sigma protein through an aminopeptidase N-dependent mechanism^30^, however, our results showed that 14-3-3ζ expression levels were increased in naive T cells co-cultured with 14-3-3ζ-overexpressing HCC cells or their visible cell culture components rather than with invisible cell culture components, suggesting that 14-3-3ζ in  TILs is influenced by 14-3-3ζ in HCC cells, at least partially mediated by visible cell culture components. Electronic microscopy and stochastic optical reconstruction microscopy (STORM)^31,32^ demonstrated that these visible components comprise mainly of exosomes and vesicles. Next, we observed that 14-3-3ζ was detectable in exosomes isolated from these visible cell culture components and that these 14-3-3ζ-containing exosomes could be swallowed by T cells in co-culture. These results as a whole suggest that 14-3-3ζ could be transmitted from HCC cells to  TILs at least partially delivered by exomoses.

However, there are still some limitations of our study that should be addressed by future studies. First, the mechanisms underlying the effects of 14-3-3ζ on  TILs function remain to be investigated; second, the possibility of other means besides exosomes by which 14-3-3ζ was transmitted from HCC cells to  TILs should not yet be excluded, such as direct contact transmission. Next, we will conduct sequencing studies to elucidate the signaling pathways involved in the functional roles of 14-3-3ζ in  TILs, and will investigate other transmission means of 14-3-3ζ.

To sum up, our study for the first time demonstrate that 14-3-3ζ impaired the anti-tumor activity of  TILs in HCC and that 14-3-3ζ could be transmitted from HCC cells to  TILs at least partially through exosomes. To the best of our knowledge, ours is the first to elucidate the functional roles of 14-3-3ζ in T cells and the underlying mechanisms of up-regulated expression of in  TILs. Our study offers a new angle to understand T cell dysfunction in HCC and may open a new avenue for the development of novel immunotherapeutic targets for HCC.

## Materials and methods

### Patients

Fresh HCC tissues, corresponding adjuvant noncancerous tissues, and HCC serum samples were collected from patients undergoing hepatectomy between Sep, 2016 and Feb, 2017. Peripheral blood samples were collected before hepatectomy. Healthy serum samples were randomly selected from patients receiving physical examination at the First Affiliated Hospital of Nanjing Medical University (Nanjing, China). The study was approved by our Institutional Ethics Committee and performed in accordance with the Helsinki Declaration and government policies. All participants have signed the written informed consent. Clinical and pathological characteristics were summarized in Table 1.

### Animals

Male C57BL/6 mice were bred and maintained under specific pathogen-free conditions in the Laboratory Animal Center of Nanjing Medical University. Immature mice were intraperitoneal injected (ip) with 25 μg/g diethylnitrosamine (DEN) at two-week old for once and subsequently challenged with 0.5 μg/g CCl4 (ip) once a week from four-week old to the end. Mice were killed under ether anesthesia at design time point and tissue samples were carefully collected. All animals received humane care and all experiments were carried out according to the guidelines outlined in the Guide for the Care and Use of Laboratory Animals.

### Cells and cell transfection

Human PBTC were cultured in RPMI-1640 medium (Invitrogen) supplemented with 10% heat-inactivated FBS, 100 U/ml penicillin, and 100 mg/ml streptomycin (Invitrogen). For lenti-virus construction, the 14-3-3ζ sequence was synthesized by GenScript Inc (China), short-hairpin RNA to interfere 14-3-3ζ in MHCC97H cells was purchased from (sc-29583, Santa Cruz, CA, USA), and then were inserted into lentiviral vector PCDH. For retrovirus construction, the 14-3-3ζ sequence was synthesized by GenScript Inc (China) and subcloned into retroviral vector pMXs. Retroviral particles were generated by 293 T cells and transfected into CD3^+^ T cells or naive T cells 24 h after activation with anti-human CD3/CD28 beads. Lentiviral particles were generated by 293 T cells, and transduced into SMCC7721 cells. Then, transductants were placed into puromycin-containing medium for 3 days before culture.

### Isolation of lymphocyte, CD3^+^, CD4^+^, CD8^+^ T cells and naive T cells

Fresh human HCC tissues (the tissue volume was recorded) were obtained and maintained in phosphate-buffered saline (PBS), then cut into pieces and filtered through a 70 μm cell strainer (BD Biosciences) into a 50 ml Falcon tube. Human peripheral blood was collected from HCC patients or healthy volunteers (10 ml each). Single-cell suspensions were softly added to the surface of 10 ml Ficoll-Paque (GE Healthcare), then centrifuged at 400 × *g* for 30 min at 20 °C according to the manufacturer’s instructions. A portion of peripheral blood mononuclear (PBMC) or tumor infiltrating lymphocytes were used to separate T cells by using anti-human CD3, CD4, or CD8 beads, isolated cells were measured by subsequent flow cytometric analysis. For naive T cells separation, PBMC was labeled with anti-human CD45RA and CD197, then naive T cells (CD45RA^+^CD197^+^) were sorted with flow cytometry on BD ARIA III (BD Biosciences).

### Stimulation of T cells

cRPMI-10 consisted of RPMI-1640 medium supplemented with 10% fetal bovine serum, antibiotic/antimycotic solution, and HEPES buffer (Sigma-Aldrich). Fetal bovine serum was from a single lot qualified for low background and high responses in flow cytometer assays.

T cells were resuspended at 1 × 10^6^ viable cells per ml in cRPMI-10 medium. For stimulation, 2 × 10^5^ cells/well were plated in polypropylene U-bottom microtiter plates in a total volume of 200 µl of RPMI-1640. They were stimulated for 8 h at 37 °C, 5% CO_2_, using 10 ng/ml phorbol 12-myristate 13-acetate (PMA, Sigma-Aldrich), 500 ng/ml ionomycin (Sigma-Aldrich) and 1 g/ml Brefeldin A (BFA, Sigma-Aldrich). After then, T cells were measured by detection of CD69 expression using flow cytometry or used for RNA extration.

Cells were stimulated with anti-CD3/CD28 beads (Thermo Fisher Scientific) at a 1:2 bead to cell ratio. Proliferation was then measured by detection of Ki67 expression using flow cytometry.

### Real time polymerase chain reaction (PCR)

Total RNA was extracted from live cells or exosomes using TRIzol reagent (Invitrogen) according to the instructions and total RNAs (500 ng) were reverse transcribed using the PrimeScript RT Master Mix (Takara). The expression levels of 14-3-3ζ was deteced by quantitative real-time PCR using the SYBR Premix Ex Taq (Takara, Dalian, China) on the ABI Prism 7900HT (Applied Biosystems). The relative expression levels of 14-3-3ζ were normalized to GAPDH (for cells) or β-Actin (for exosomes). The reactions were incubated in a 384-well optical plate at 95 °C for 30 s, followed by 40 cycles of 95 °C for 5 s and 60 °C for 30 s. Related primer sequence was provided in Table S1.

### Immunohistochemistry and western blot

For IHC, tissues were fixed in 4% paraformaldehyde overnight at 4 °C, processed by paraffin embedding, then sectioned into 5 mm^3^ slices, and dewaxed. Antigen retrieval was performed by heating the slides in the autoclave for 3 min using citrate buffer (pH6.0). The slides were incubated overnight at 4 °C with the following primary antibodies (diluted at 1/1000): anti-14-3-3ζ antibody (Cell signaling technology). Immunodetection was performed with a UltraSensitiveTM SP(Mouse/Rabbit) IHC Kit (Fuzhou Maixin Biotech.). Images were acquired using a microscope (Nikon, ECLIPSE 50i) equipped with software NIS Elements v4.0, and average IOD was obtained by randomly collecting and analyzing five fields per slide using Image-Pro Plus software v.5.0. Detection for each index was performed for at least three times.

For western blot assay, total proteins were extracted from cells or exosomes using radio-immunoprecipitation assay buffer plus fresh protease and phosphatase inhibitors (Beyotime), then quantified with the Bradford assay (Bio-Rad Laboratories). Equal amounts of protein samples (30 μg) were loaded to each lane, then separated by sodium dodecyl sulfate polyacrylamide gel electrophoresis (SDS–PAGE) and subsequently transferred to a polyvinylidene fluoride membrane. Antibodies against 14-3-3ζ (Cell signaling technology), human reduced glyceraldehyde-phosphate dehydrogenase (GAPDH) (Cell Signaling Technology) and β-actin (Cell Signaling Technology) were used in immunoblotting. The integrated density of the bands was quantified using ImageJ software (NIH).

### Exosomes isolation

Exosomes in supernatant were collected from HCC cells cultured for 48-72 h. The supernatant of cell culture medium was collected, then centrifuged at 800 × *g* for 5 min, followed by centrifugation of 2000 × *g* for 10 min to remove cellular debris. Then, the media was filtered through a 0.2 μm pore strainer (Syringe filter), and then ultracentrifuged at 100,000 × *g* for 2 h at 4 °C. The pellet was washed with 35 ml 1 PBS, followed by a second step of ultracentrifugation at 100,000 × *g* for 2 h at 4 °C. Afterwards, the supernatant was discarded.

For serum exosomes isolation, serum was diluted in 1 × PBS and filtered through a 0.2 μm filter, then ultracentrifuged at 150,000 × g overnight at 4 °C. Next, the pellet was washed in 10 ml 1 × PBS followed by ultracentrifugation at 150,000 × *g* at 4 °C for 2 h. The pelleted (exosomes) was collected. Exosomes resuspended in 500 μl of Trizol were for RNA extraction, resuspended in 250 μl of lysis buffer (8 M Urea/2.5%SDS, 5 μg/ml leupeptin, 1 μg/ml pepstatin and 1 mM phenylmethylsulphonyl fluoride) for protein extraction or re-suspended in PBS for immunofluorescence staining.

### Enzyme-linked immuno sorbent assay (ELISA) assay

Exosome suffering freezing and freeze-thaw cycles were used to extract protein for ELISA assay, which was performed with an ELISA Kit for 14-3-3ζ (R&D Systems Inc., Minneapolis, USA) according to the instruction.

### Immunofluorescence

For CD63 immunofluorescence label of exosomes, 4 μl of exosome specimens was diluted in 400 μl of PBS buffer mixed with 1 μl of primary antibody (1 mg/ml rabbit anti-CD63, Thermofisher Scientific). Samples the were incubated for 2 h at room temperature. For the blank control, the PBS buffer instead of the primary antibody was used. Then, the reaction solution was treated with ultrafiltration (50 Kda) at 6000 rpm for 20 min. The precipitation was collected and suspended in PBS. Next, 1 μL of secondary antibody (2 mg/ml Alexa Fluor 647 labeled Goat anti-Rabbit IgG, ab150079, Abcam) was added. The mixture was vigorously stirred for 1 h at room temperature and then treated with ultrafiltration (50 Kda) at 6000 rpm for 20 min to remove excess reagents. The precipitation was collected and resuspended in 400 μL of PBS for stochastic optical reconstruction microscopy (STORM).

To immunofluorescence label 14-3-3ζ, cells were fixed in fresh acetone followed by permeabilization in 0.2% Triton X-100/0.5% normal goat serum/PBS, then incubated with primary antibody against 14-3-3ζ (Cell signaling technology) and secondary antibodies labeled with FITC. DAPI was used for nuclear staining. The cells were visualized using Confocal Laser Scanning Microscope (Leica TCS SP8).

### Uptake of exosomes

Exosomes were incubated in 1uM 1,1′-dioctadecyl-3,3,3′3′-tetramethylindocarbocyanine perchlorate (DiI, Thermofisher Scientific) for 20 min, followed by an additional round of PBS wash. PBS was then used to resuspend the exosomes for cell treatment. Cells were co-cultured with DiI-labeled exosomes for 2 h. To simultaneously label 14-3-3ζ, the immunofluorescence experiment followed a similar procedure as described above.

### Flow cytometry assay

Isolated T cells were collected by centrifugation (330 × *g*, 5 min at 4 ℃) and cell counts were determined by using a Countess Counter (Invitrogen). For surface staining, cells were resuspended in PBS (with 2% FBS) containing the antibody, incubated on ice for 30 min, washed in PBS (with 2% FBS), and analyzed using flow cytometry. Flow cytometry assay was performed on CANTO II (BD Biosciences). For intracellular molecule detection, fixation and permeabilization buffers (eBioscience) were used according to the instructions. Data were collected on BD CANTO II (BD Biosciences) with FACSCanto software version 2.1 (BD). Antibodies used were described in Table S2.

### Statistical analysis

All statistical analyses were performed using SPSS 18.0 (SPSS Inc, Chicago, IL, USA) software and presented with the GraphPad prism software (GraphPad Software, San Diego, CA, USA). FlowJo 7.6.1, https://www.flowjo.com/solutions/flowjo) was used to analyze and output the data of flow cytometry assay. Results of quantitative real-time PCR were expressed as mean ± S.E.M. Student’s *t* test. In all cases, *P* < 0.05 was considered significant.

## Electronic supplementary material

Fig S1

Fig S2

Fig S3

Fig S4

Fig S5

Table S1

Table S2
