# Supplementary figures and images for: 14-3-3ζ delivered by hepatocellular carcinoma-derived exosomes impaired anti-tumor function of tumor-infiltrating T lymphocytes
Source: Cell Death Dis. 2018 Feb 7;9(2):159. doi: 10.1038/s41419-017-0180-7 (PMC5833352; doi:10.1038/s41419-017-0180-7)

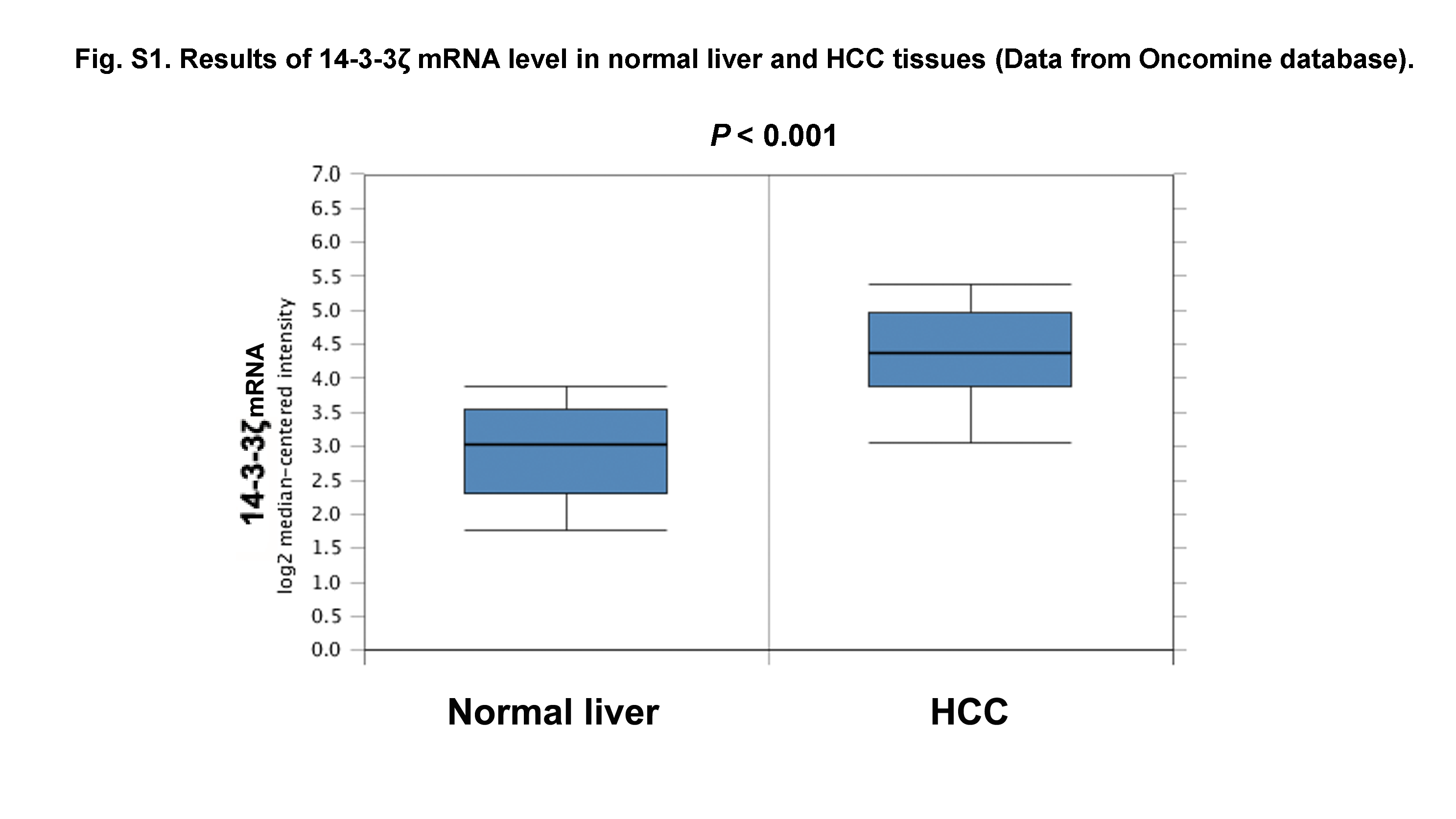

Supplement: Supplementary file 1 — Fig S1 [file 41419_2017_180_MOESM1_ESM.tiff]

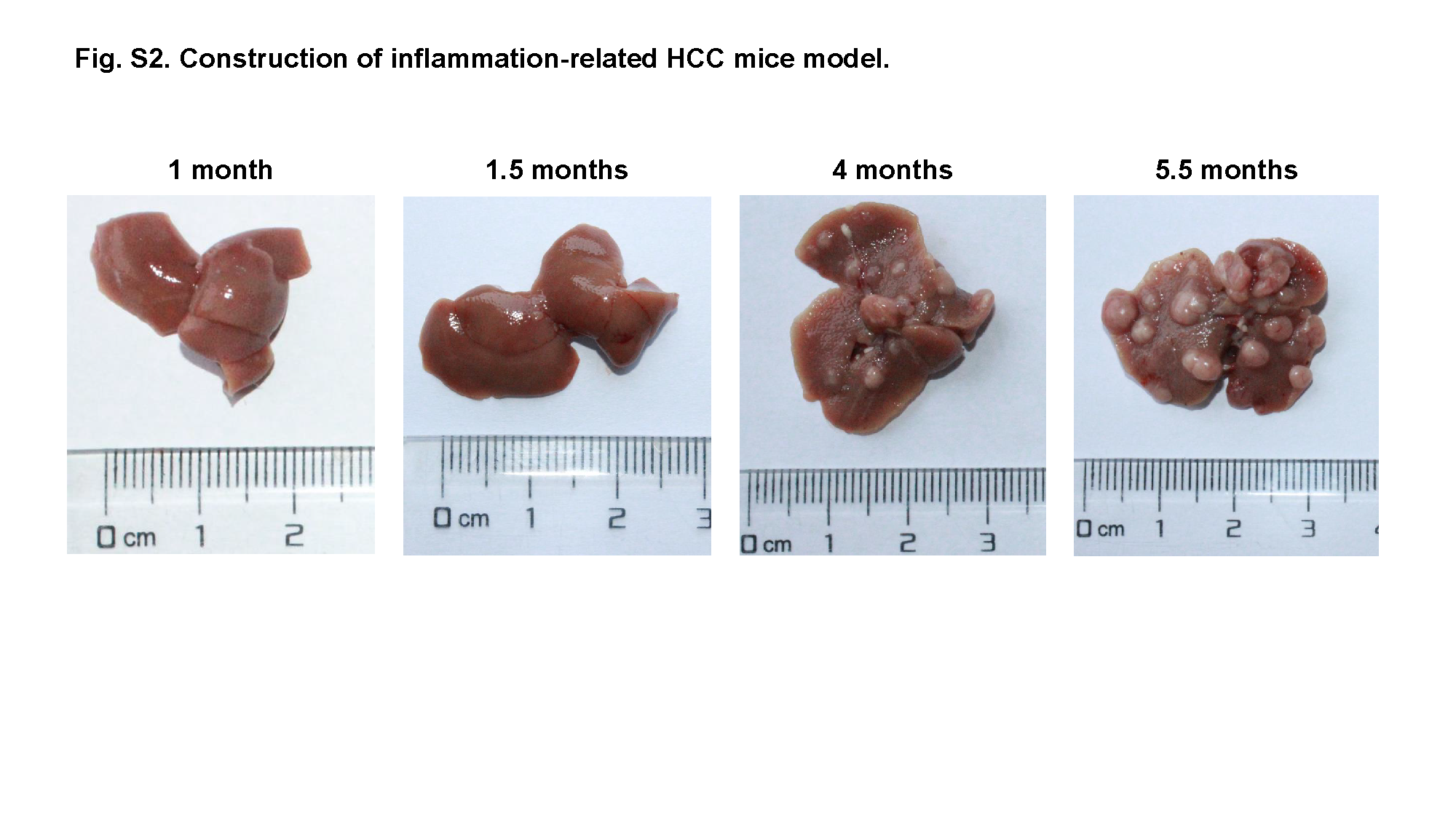

Supplement: Supplementary file 2 — Fig S2 [file 41419_2017_180_MOESM2_ESM.tiff]

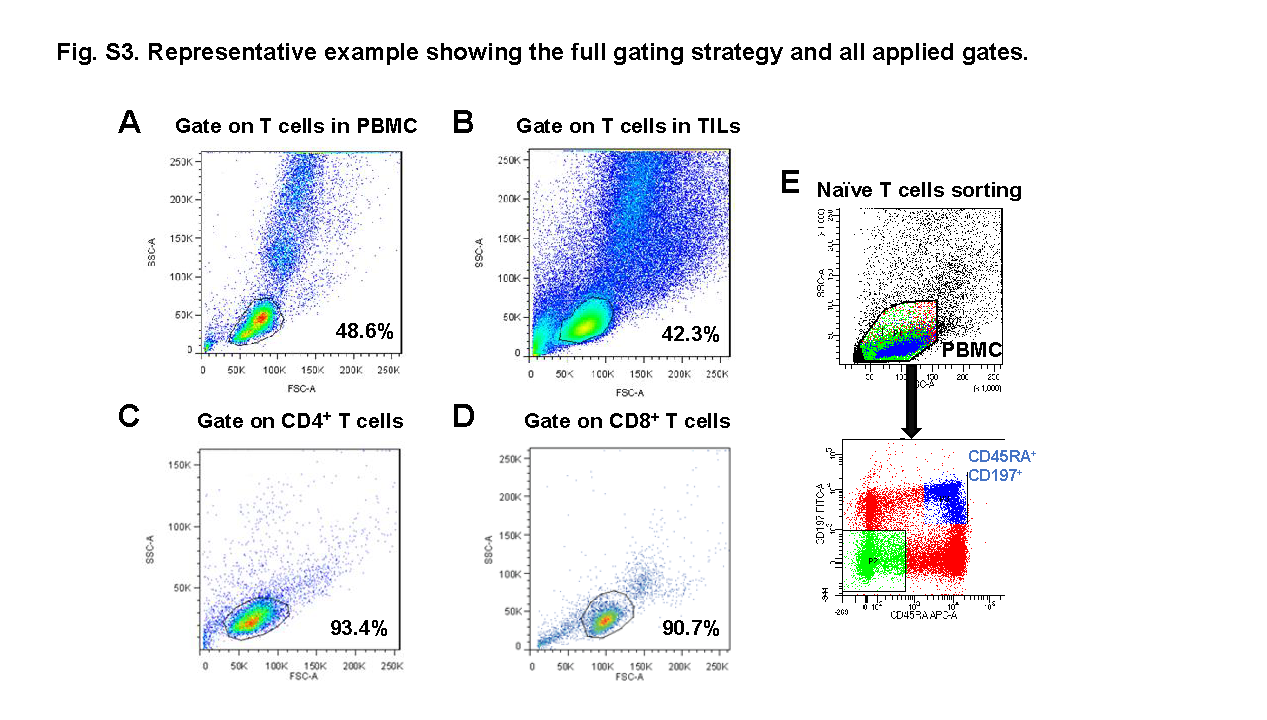

Supplement: Supplementary file 3 — Fig S3 [file 41419_2017_180_MOESM3_ESM.tiff]

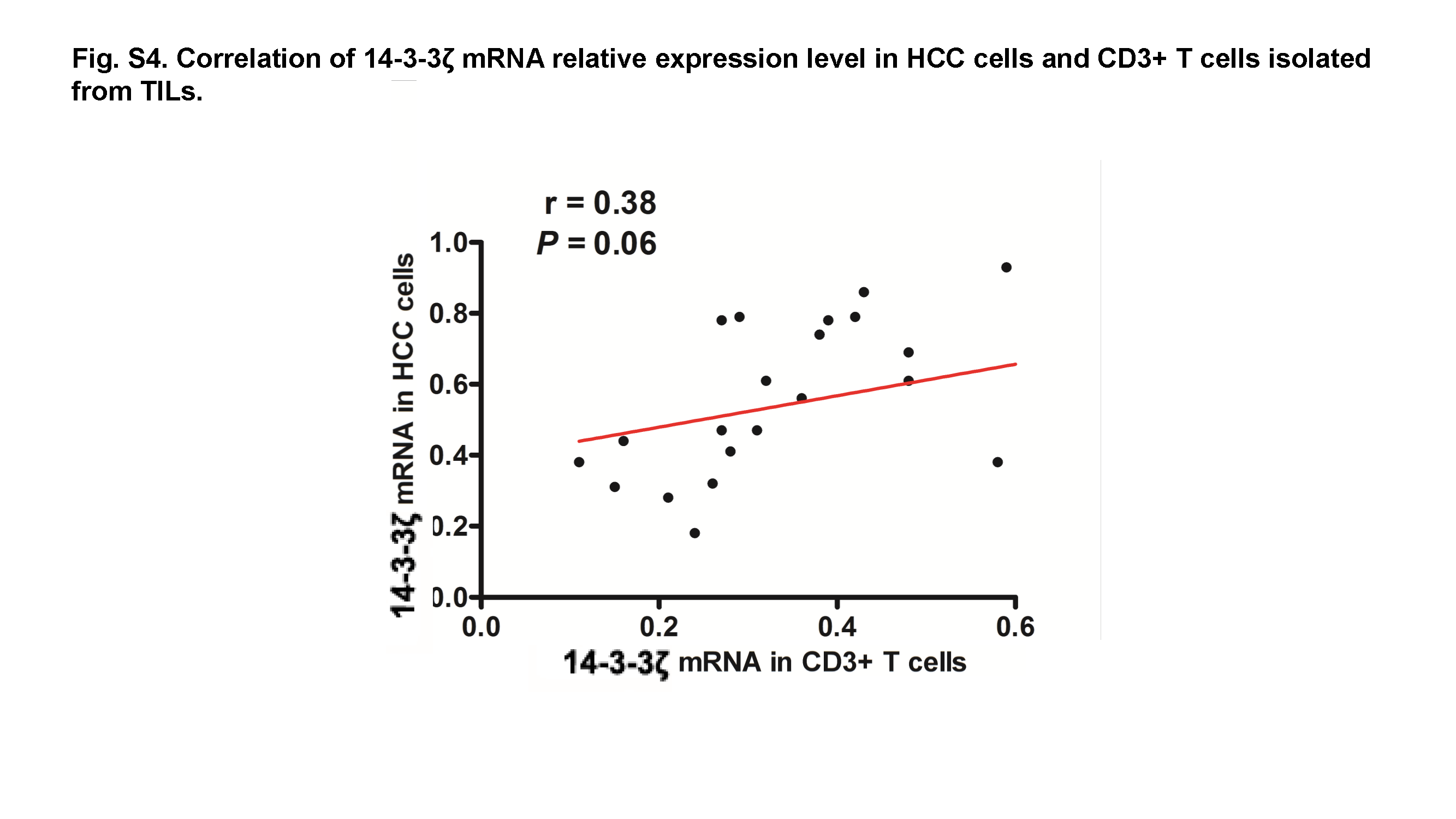

Supplement: Supplementary file 4 — Fig S4 [file 41419_2017_180_MOESM4_ESM.tiff]

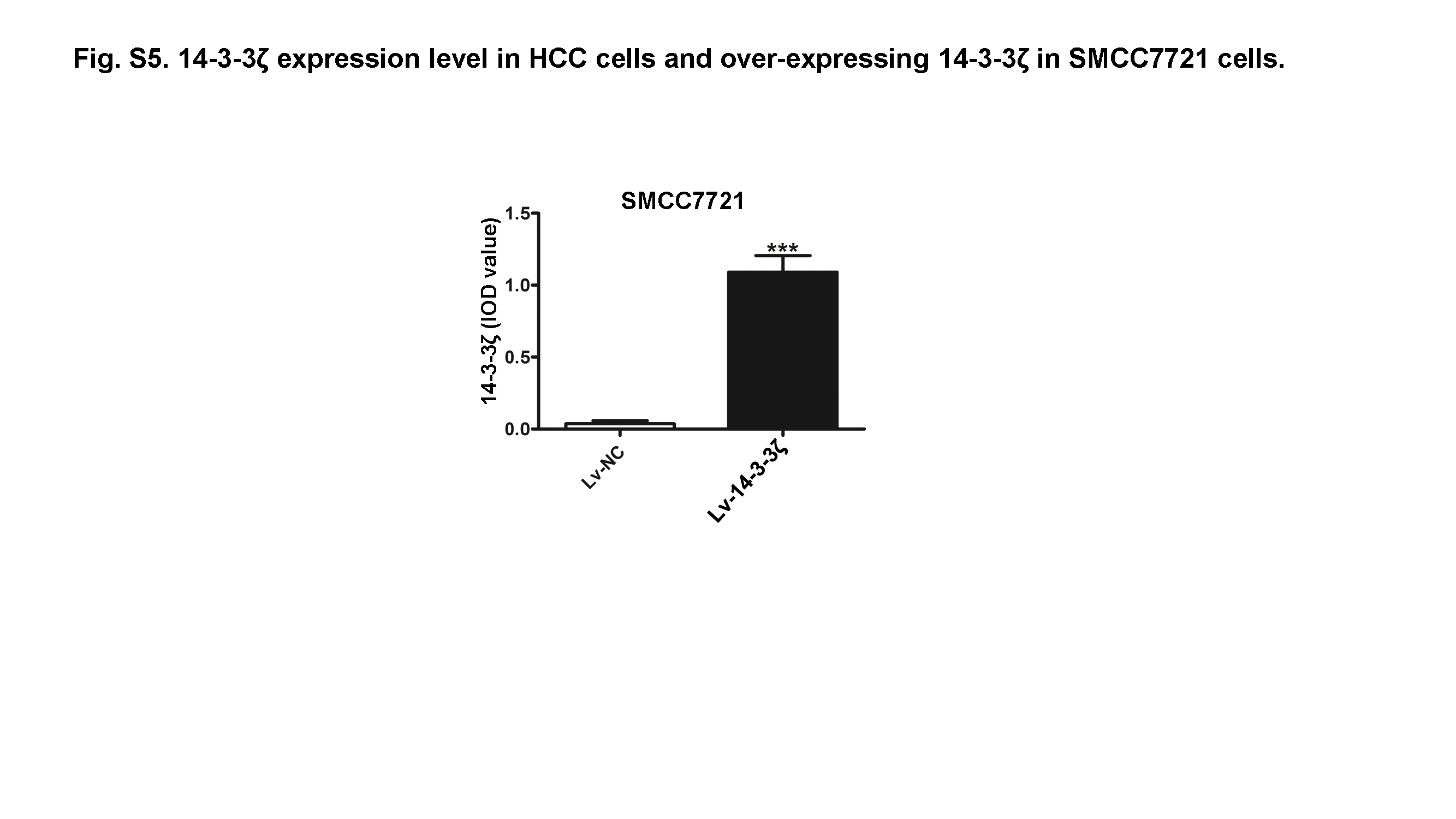

Supplement: Supplementary file 5 — Fig S5 [file 41419_2017_180_MOESM5_ESM.tiff]
