# Supplementary material for: 14-3-3ζ delivered by hepatocellular carcinoma-derived exosomes impaired anti-tumor function of tumor-infiltrating T lymphocytes
Source: Cell Death Dis. 2018 Feb 7;9(2):159. doi: 10.1038/s41419-017-0180-7 (PMC5833352; doi:10.1038/s41419-017-0180-7)
Supplement: Supplementary file 6 — Table S1 [file 41419_2017_180_MOESM6_ESM.docx]

**Table S1.** Primers used for real-time PCR.

| Genes | Forward(5’~3’) | Reverse(5’~3’) |
| --- | --- | --- |
| IFN-γ | TGATTTCCTGGCCTTCATTC | ACAGAGGGCCAAAAAGGTCT |
| GAPDH | GCCTCGTCCCGTAGACAAAA | GATGGGCTTCCCGTTGATGA |
| IL-12 | TACTAGAGAGACTTCTTCCACAACAAGAG | TCTGGTACATCTTCAAGTCCTCATAGA |
| β-actin | CATGTACGTTGCTATCCAGGC | CTCCTTAATGTCACGCACGAT |
| 14-3-3ζ | TGTAGGAGCCCGTAGGTCATC | GTGAAGCATTGGGGATCAAGA |

Other primers utilized for real-time PCR analysis were recommended by PrimerBank (http://pga.mgh.harvard.edu/primerbank/index.html)
