# Supplementary material for: 14-3-3ζ delivered by hepatocellular carcinoma-derived exosomes impaired anti-tumor function of tumor-infiltrating T lymphocytes
Source: Cell Death Dis. 2018 Feb 7;9(2):159. doi: 10.1038/s41419-017-0180-7 (PMC5833352; doi:10.1038/s41419-017-0180-7)
Supplement: Supplementary file 7 — Table S2 [file 41419_2017_180_MOESM7_ESM.docx]

**Table S2.** Regents used in experiments.

| **Regents** | **Clone** | **Application** | **Supplier** |
| --- | --- | --- | --- |
| FITC anti-human CD69 | 310904 | Flowcytometry | BioLegend |
| PE anti-human IFN-γ | 502515 | Flowcytometry | BioLegend |
| AlexaFluor488 anti-human FOXp3 | 320012 | Flowcytometry | BioLegend |
| FITC anti-human Ki67 | 11-5699-42 | Flowcytometry | eBioscience |
| Live/Dead Fixable Red Dead Stain | L10120 | Flowcytometry | Thermofisher Scientific |
| Brilliant Violet 421 anti-human CD4 | 317434 | Flowcytometry | BioLegend |
| APC anti-human CD8 | 344722 | Flowcytometry | BioLegend |
| FITC anti-human CD223 (LAG-3) | 369307 | Flowcytometry | BioLegend |
| PE anti-human CD279 (PD1) | 130-096-164 | Flowcytometry | Miltenyi |
| APC anti-human TIM3 | 130-098-936 | Flowcytometry | Miltenyi |
| Brilliant Violet 421 anti-human CTLA4 | 369606 | Flowcytometry | BioLegend |
| APC anti-human CD45RA | 304112 | Flowcytometry cells sorting | BioLegend |
| FITC anti-human CD197 (CCR7) | 353216 | Flowcytometry cells sorting | BioLegend |
| Exosome – Human CD63 Isolation/Detection | 10606D | Immunofluorescence | Thermofisher Scientific |
| CellTracker™ CM-DiI | C7000 | Immunofluorescence | Thermofisher Scientific |
| DAPI | D1306 | Immunofluorescence | Thermofisher Scientific |
| CD4 MicroBeads, human | 130-045-101 | Cells sorting | Miltenyi |
| CD8 MicroBeads, human | 130-045-201 | Cells sorting | Miltenyi |
| CD3 MicroBeads, human | 130-050-101 | Cells sorting | Miltenyi |
| Dynabeads Human T-Activator CD3/CD28 | 11131D | T cell activation | Thermofisher Scientific |
| 14-3-3Ζ  (14-3-3 ζ/δ) | 7413 | Western Blot, Immunohistochemistry,  Immunofluorescence | CST |
| Mouse Anti-rabbit IgG | 3678 | Western Blot | CST |
| UltraSensitiveTM SP(Mouse/Rabbit)IHC Kit | KIT-9710 | Immunohistochemistry,  Immunofluorescence | Fuzhou Maixin Biotech. |
